# Brain functional connectivity abnormalities in attention‐deficit hyperactivity disorder

**DOI:** 10.1002/brb3.583

**Published:** 2016-09-28

**Authors:** Richard B. Silberstein, Andrew Pipingas, Maree Farrow, Florence Levy, Con K. Stough, David A. Camfield

**Affiliations:** ^1^ Centre for Human PsychopharmacologySwinburne UniversityHawthornVic.Australia; ^2^ Neuro‐Insight Pty LtdMelbourneVic.Australia; ^3^ Wicking Dementia Research & Education CentreUniversity of TasmaniaHobartTas.Australia; ^4^ Prince of Wales Hospital and School of PsychiatryUniversity of New South WalesSydneyNSWAustralia

**Keywords:** brain functional connectivity, default mode network, steady state visually evoked potential

## Abstract

**Introduction:**

Recent evidence suggests that attention‐deficit hyperactivity disorder (ADHD) is associated with brain functional connectivity (FC) abnormalities.

**Methods:**

In this study, we use steady‐state visually evoked potential event‐related partial coherence as a measure of brain FC to examine functional connectivity differences between a typically developing (TD) group of 25 boys and an age/IQ‐matched group of 42 drug naive boys newly diagnosed with ADHD (ADHD group). Functional connectivity was estimated while both groups performed a low‐demand reference task and the A‐X version of the continuous performance task (CPT A‐X).

**Results:**

While the TD and ADHD groups exhibited similar prefrontal FC increases prior to the appearance of the target in the reference task, these groups demonstrated significant FC differences in the interval preceding the appearance of the target in the CPT A‐X task. Specifically, the ADHD group exhibited robust prefrontal and parieto‐frontal FC increases that were not apparent in the TD group.

**Conclusion:**

The FC differences observed in the ADHD group are discussed in the context of inadequate suppression of cortical networks that may interfere with task performance.

## 1 Introduction

Attention‐deficit hyperactivity disorder (ADHD) is a disorder affecting an estimated 3–6% of children and is characterized by symptoms of inattention, impulsivity, and/or hyperactivity (Brown & Cooke, 1994). Stimulant medications, such as methylphenidate (MPH), which inhibit the reuptake of dopamine (DA) and noradrenaline (NA), have had a long history of use in the treatment of ADHD; the reason for their effectiveness being that catecholamine transmission in fronto‐striato‐cerebellar circuits is typically impaired in ADHD (Del Campo, Chamberlain, Sahakian, & Robbins, 2011). In recent years, much progress has been made in better understanding how ADHD symptoms are related to dysfunction in various components of frontostriatal circuitry (dorsolateral prefrontal cortex [DLPFC], ventrolateral prefrontal cortex [VLPFC], dorsal anterior cingulate cortex [dACC], and striatum) (Bush, Valera, & Seidman, 2005; Silberstein et al., 1998), as well as the parietal cortex, brainstem, and cerebellum (Valera, Faraone, Murray, & Seidman, 2007). However, research addressing the question of disordered functional connectivity (FC) in ADHD is a relatively recent development.

One common approach to this is the use of fMRI to determine the resting‐state FC (fMRI‐RSFC). The fMRI‐RSFC is derived from the correlations in the time‐varying BOLD signal between various brain sites during a no‐task or resting state. Regions exhibiting a high correlation (positive or negative) are deemed to have a functional relationship (Raichle et al., 2001). Several fMRI‐RSFC studies of ADHD have focused on abnormalities, especially reduced connectivity within the default mode network (DMN) and differences in the FC between the DMN and the networks that become more active during a cognitive task or task‐positive networks. The DMN is a network comprising a number of regions including the ventrolateral and ventromedial prefrontal cortex, the posterior cingulate cortex (PCC), the precuneus and the inferior parietal lobe. The DMN is most active when awake subjects are resting and not engaged in a cognitive task (Raichle et al., 2001; Buckner et al., 2008). In general, the DMN becomes less active during a cognitive task when other task‐positive networks become more active although there are important exceptions to this rule (see Gerlach, Spreng, Gilmore, & Schacter, 2011; Gerlach, Spreng, Madore, & Schacter, 2014). One network frequently active during cognitive tasks is a frontoparietal network also known as the “executive control circuit”. This comprises lateral frontal poles, anterior cingulate cortex (ACC), dorsolateral prefrontal cortex, anterior prefrontal cortex, lateral cerebellum, anterior insula, and inferior parietal cortex (Liston, Cohen, Teslovich, Levenson, & Casey, 2011). Activity in the executive control circuit is negatively (or anti) correlated with the DMN in that high activity in one is associated with reduced activity in the other. Lapses in sustained attention are associated with DMN activity during such tasks. A reduced negative correlation between the DMN and task active networks has been reported in ADHD (Castellanos et al., 2008; Christakou et al., 2013; Konrad & Eickhoff, 2010; Liston et al., 2011; Sun et al., 2012). Sonuga‐Barke and Castellanos (2007) suggest that the inattentiveness observed in ADHD could be due to inadequate suppression of the DMN and its increased activity is associated with the intrusion of thoughts unrelated to the task or “day dreaming” (Fassbender et al., 2009; Kucyi & Davis, 2014). A meta‐analysis of 55 (39 children studies) ADHD fMRI task‐based studies indicated that the most consistent findings were that compared to controls, the ADHD groups exhibited hyperactivity in the DMN and hypoactivity in the task‐positive networks, such as the frontoparietal and ventral attentional networks during cognitive tasks (Cortese et al., 2012).

**Figure 1:**
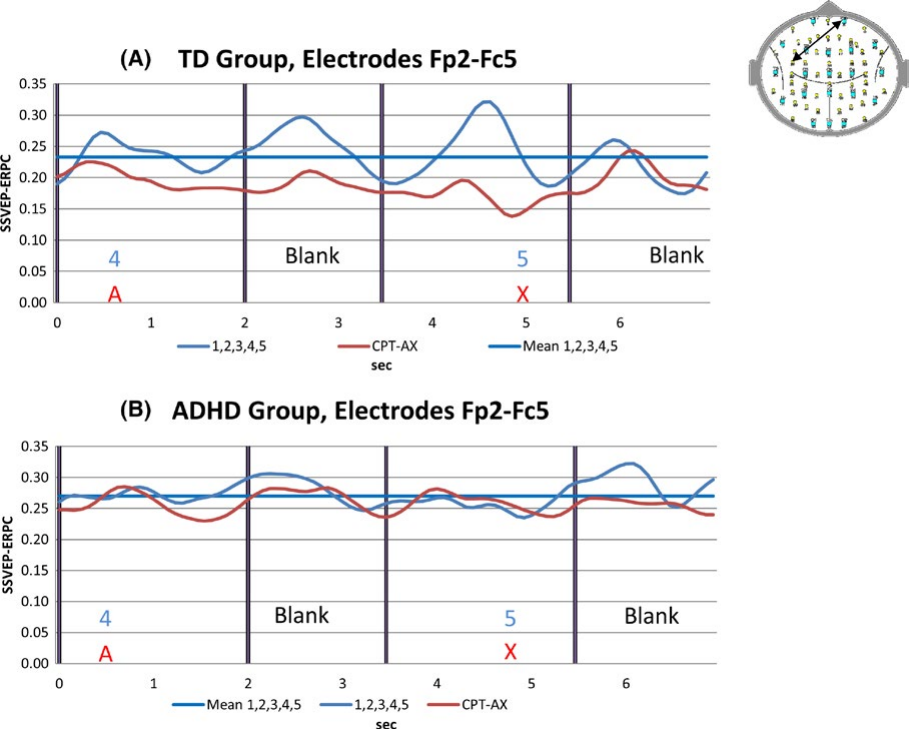
(A) TD group pooled SSVEP event‐Related partial coherence (SSVEP‐ERPC) or functional connectivity (FC) for electrode pair Fp2‐Fc5 for the reference task (blue trace) and for the continuous performance task (CPT) A‐X task (red trace). The solid blue line indicates the mean value of the reference task FC for this electrode pair. (B) Attention‐deficit hyperactivity disorder (ADHD) group pooled functional connectivity for electrode pair Fp2‐Fc5 for the reference task (blue trace), CPT A‐X task (red trace) and the mean of the reference task FC for the electrode pair (solid blue line). Note the reduction in FC during the CPT A‐X task compared to the reference task in the TD group. By contrast, no such FC reduction is apparent in the ADHD group

Electroencephalogram (EEG) studies have also indicated evidence of reduced inhibition of the DMN during cognitive tasks in ADHD. Helps, James, Debener, Karl, and Sonuga‐Barke (2008), Helps, Broyd, James, Karl, and Sonuga‐Barke (2009), and Helps et al. (2010) used DC‐EEG recording technology to examine the behavior and topography of delta EEG (1.5–4 Hz) very low‐frequency EEG rhythms in the range of 0.06–1.5 Hz while subjects were in a resting state and while performing a visual vigilance task. They found that only the 0.06–0.2 Hz component exhibited DMN behavior in that it was attenuated when subjects performed the vigilance task compared to the resting state. Furthermore, the level of reduction in this component varied with the level of ADHD symptomatology. Subjects scoring high on symptoms of inattention showed less task‐related attenuation of this component (Broyd, Helps, & Sonuga‐Barke, 2011; Helps et al., 2010). Interestingly, the 0.06–0.2 Hz component exhibiting DMN properties was most prominent at prefrontal, frontal, and parietal locations (see Fig. 1, Helps et al., 2010). Broyd et al. (2011) employed LORETA software as an inverse imaging technique to identify the cortical generators of the 0.06–0.2 Hz generators. They found that the frontal and prefrontal components originated in the medial prefrontal regions including the cingulate gyrus (BA 23, BA24, BA32) as well as the superior frontal gyrus (BA8) and medial prefrontal gyrus (BA8), while the parietal components was found to originate in the vicinity of the PCC and precuneus; all regions identified as part of the DMN (Buckner, Andrews‐Hanna, & Schacter, 2008).

Although studies of FC using fMRI provide important insights into dysfunctional connectivity in ADHD, there is an important limitation to this technique that needs to be acknowledged. fMRI, as an indirect measure of brain activity, measures magnetic changes associated with fluctuations in the fMRI blood oxygen level‐dependent (BOLD) signal, rather than neural activity itself (Ritter & Villringer, 2006). For this reason, the temporal resolution of this technique is limited by the hemodynamic response function, and is typically of the order of several seconds (Kim, Richter, & Ugurbil, 1997). The implication of this limited temporal resolution is that transient changes in FC which occur at higher frequencies cannot be captured. This limitation is particularly pertinent for the study of ADHD in light of the fact that many of the cognitive processes of interest in this disorder, such as selective attention and working memory, have been found to be mediated by FC in the alpha, beta, and theta band (Bressler & Menon, 2010; Bressler & Richter, 2015; Silberstein, 2006; Von Stein & Sarnthein, 2000).

In contrast to fMRI, the recording of the EEG coherence provides superior temporal resolution (at the price of lower spatial resolution) for the investigation of transient changes in connectivity that may occur during cognitive processing. In addition to traditional methods of EEG coherence, the measurement of changes in phase coupling between brain sites during cognitive processes can provide valuable information regarding the timing of communication between these regions (Sauseng & Klimesch, 2008). One evoked potential methodology used to measure FC makes use of the steady‐state visually evoked potential (SSVEP) in response to a diffuse flicker (see Silberstein, Cadusch, Nield, Pipingas, & Simpson, 1996; Silberstein, Nunez, Pipingas, Harris, & Danieli, 2001). We have previously shown that cognitive tasks performed while subjects are simultaneously exposed to an ongoing peripheral spatially diffuse 13 Hz visual flicker are associated with task‐dependent changes in the amplitude and phase of the 13 Hz sinusoidal evoked potential or 13 Hz SSVEP (Silberstein, 1995; Silberstein et al., 1998). The methodology, termed steady‐state topography (and in some earlier papers steady‐state probe topography) has been used to examine the scalp topography of SSVEP amplitude and phase variations associated with a range of cognitive tasks in typical and patient populations (Silberstein, Line, Pipingas, Copolov, & Harris, 2000; Silberstein et al., 2001). One important advantage of steady‐state topography is the high SSVEP signal‐to‐noise ratio is in turn associated with a high resistance to most common EEG artifacts, such as EOG, blink and movement artifacts, mains interference, and EMG (Gray, Kemp, Silberstein, & Nathan, 2003; Silberstein, 1995).

In the current study, we measure changes in brain FC using a methodology termed *SSVEP Event‐Related Partial Coherence* (SSVEP‐ERPC) to derive a measure of the SSVEP phase coherence between scalp recording sites. The SSVEP‐ERPC provides a measure of the degree to which the SSVEP phase differences between electrode pairs remain stable across trials once the common contribution of the stimulus eliciting the SSVEP has been removed (Silberstein, 2006; Silberstein, Danieli, & Nunez, 2003; Silberstein, Song, Nunez, & Park, 2004). The SSVEP‐ERPC technique has been previously used by our laboratory to investigate patterns of FC associated with mental rotation (Silberstein, 2006; Silberstein et al., 2003) as well as performance on Raven's advanced progressive matrices (Silberstein et al., 2004). In both these studies, FC topography was found to change dramatically over the time course of the trial, and processing speed was found to form highly significant correlations with connectivity at different stages of task processing. We interpret the SSVEP‐ERPC as a measure of FC and the terms, *functional connectivity* and SSVEP‐ERPC will be used interchangeably throughout this article.

In this study, the SSVEP‐ERPC technique is applied for the first time to the study of FC in a sample of stimulant drug medication naive boys newly diagnosed with ADHD (ADHD group). Functional connectivity in the ADHD group is compared with patterns of connectivity in an IQ‐matched and age‐matched typically developing group (TD group) while performing the A‐X variant of a continuous performance task (CPT A‐X). The CPT A‐X is a neuropsychological test that has been found to be highly sensitive to attentional disturbance in ADHD (Riccio, Reynolds, Lowe, & Moore, 2002).

We hypothesize that the ADHD group will exhibit differences in FC associated with elevated activity of neural networks that may interfere with task performance, such as but not limited to the DMN. For the sake of brevity, we will refer to networks that interfere with task performance as the task interfering network (TIN). Specifically, in the TD group we would expect that FC associated with TINs, such as the DMN, should be reduced in the CPT A‐X task compared to TIN activity during the performance of a less demanding reference task. By comparison, in the ADHD group, we expect to see less attenuation of TIN FC during the CPT A‐X task when compared to FC in the reference task.

## 2 Methods

### 2.1 Participants

The TD group consisted of 25 males with a mean age of 10.83 years (*SD* = 1.74 years) and a mean IQ of 110.96 (*SD* = 6.02). The ADHD group consisted of 42 males with a mean age of 10.04 years (*SD* = 2.00 years) and a mean IQ of 107.62 (*SD* = 9.48). One‐way ANOVA confirmed that the control and ADHD groups were not significantly different in either age (*F*(1, 66)  = 2.687, *p *> .05) or IQ (*F*(1, 66)  = 2.497, *p *> .05). The ADHD group comprised stimulant drug naive participants who met eight or more DSM‐IV criteria for ADHD and were newly diagnosed. All ADHD participants were recruited through the Royal Children's Hospital, Melbourne, while control participants were recruited through advertisements placed in the wider community. The study was approved by the Human Research Ethics Committee of Swinburne University as well as the Australian National Health and Medical Research Council Twin Registry.

### 2.2 Cognitive tasks

All participants first performed a low‐demand visual vigilance task which served as a reference task followed by the CPT A‐X task. In the reference task, participants viewed a repeated presentation of the numbers 1, 2, 3, 4, and 5 and were required to press a microswitch on the appearance of the 5. In the CPT A‐X, task participants were required to respond on the unpredictable appearance of an X that had been preceded by an A. In all tasks, the numbers or letters remained on the screen for 2 s and were followed by a blank screen for 1.5 s. All letters and numbers where white, and presented on a black screen. The ratio of targets to nontargets was 1:4 and the task duration was 280 s. Reaction time was recorded to an accuracy of 1 ms. For all tasks, a correct response to a target was defined as one that occurred no <100 ms and no more than 1.5 s after the appearance of the target (5 or an X preceded by an A). Any responses outside the “correct” time intervals were defined as errors of commission, or false alarms, while failure to respond in the correct interval was defined as an error of omission.

The cognitive tasks were presented on a computer monitor. Each letter and number subtended a horizontal and vertical angle of approximately 1.0° when viewed by subjects from a fixed distance of 1.3 m. The stimulus used to evoke the SSVEP was a spatially diffuse 13‐Hz sinusoidal flicker subtending a horizontal angle of 160° and a vertical angle of 90°, which was superimposed on the visual fields. This flicker was present throughout the task and special goggles enabled subjects to simultaneously view the cognitive task and the sinusoidal flicker. The modulation depth of the stimulus when viewed against the background was 45%.

### 2.3 SSVEP recording/processing

Brain electrical activity was recorded from 64 scalp sites that included all international 10–20 positions, with additional sites located midway between 10–20 locations. The specific locations of the recording sites have been previously described (Silberstein, 2006). The average potential of both earlobes served as a reference and a nose electrode served as a ground. Brain electrical activity was amplified and bandpass filtered (3 dB down at 0.1 Hz and 30 Hz) before digitization to 16‐bit accuracy at a rate of 400 Hz. The major features of the signal processing have been described (Silberstein, 2006; Silberstein et al., 2003). Briefly, the SSVEP was determined from the 13‐Hz Fourier coefficients evaluated over 10 stimulus cycles at the stimulus frequency of 13 Hz, thus yielding a temporal resolution of 0.77 s. The 10‐cycle evaluation period was shifted one stimulus cycle and the coefficients were recalculated for this overlapping period. This process was continued until the entire 280 s of activity was analyzed. An identical procedure was applied to data recorded from all 64 recording sites.

### 2.4 SSVEP event‐related partial coherence

For each subject, the SSVEP‐ERPC was calculated for all 2016 distinct pairs of electrodes averaged across all correct responses in the reference and CPT A‐X tasks. The SSVEP‐ERPC is a measure of the partial coherence between electrode pairs at the stimulus frequency eliciting the SSVEP and is based on a modification of an approach first described by Andrew and Pfurtscheller (1996) (Silberstein et al., 2003).

Partial coherence varies between 0 and 1, and like coherence is a normalized quantity that is not determined by the SSVEP amplitude at either electrode site. Electrode pairs with high partial coherence indicate relatively stable SSVEP phase differences between electrode pairs across trials. This occurs even though SSVEP phase differences between each of the electrodes and the stimulus may be variable across trials and is equivalent to the removal of the common contribution from the SSVEP stimulus. This means that high SSVEP partial coherence between electrodes reflects a consistent synchronization between electrodes at the stimulus frequency and is not simply a consequence of two unrelated regions increasing their response to the common visual flicker. Such synchronization reflected in the SSVEP‐ERPC is thought to reflect FC between the relevant regions and as mentioned earlier, we will use the terms “SSVEP‐ERPC” and “functional connectivity” (FC) interchangeably.

For the reference task, FC was determined during the 7.0 s interval that comprised an initial 2.0 s period where the number “4” was displayed followed by a 1.5 s blank screen that was in turn followed by the 2.0 s appearance of the “5” followed by another 1.5 s blank screen. The equivalent FC was calculated for the “A”, “blank”, “X”, blank, intervals of the CPT A‐X. Only FC data associated with correct Ref and CPT A‐X trials was used.

### 2.5 Statistical considerations

To examine the differences in FC between the TD and ADHD groups, we conducted an independent Student's *t*‐test for each point in time for all of the 2016 distinct electrode pairs while participants performed the CPT A‐X task. We then calculated the number of electrode pairs where the magnitude of Student's *t* |*t*| is equal to or exceeds a specified threshold level and displayed the electrode pairs where the |*t*| threshold was equaled or exceeded. For this unpaired comparison comprising 67 participants in total (*df* = 65), we used a threshold |*t*| value of |*t*| ≥ 3.45 corresponding to *p *≤ .001.

To examine the difference in FC during the reference or the CPT A‐X task compared to the mean of the reference task (*meanRef*), we calculated the one‐sample Student's *t*‐test for each point in time for all of *the* 2016 distinct electrode pairs. We then calculated the number of electrode pairs where the magnitude of Student's *t* |*t*| is equal to or exceeds a specified threshold level and displayed the electrode pairs where the |*t*| threshold was equaled or exceeded. For the TD group, we select the threshold to be |*t*| ≥ 2.78 which corresponds to *p *≤ .01 for 25 participants and a *Z*‐score of *Z *≥ 0.56. Positive and negative *t*‐values corresponding to the positive or negative threshold are displayed separately in terms of the number and location of electrode pairs. Graphs illustrating the number of electrode pairs where *t* ≥ threshold (positive deviations) are illustrated in red while the converse (negative deviation, ‐*t *≤ ‐threshold) are illustrated in blue and such graphs are termed *Student t‐frequency curves*.

In this study, we set the threshold |*t*| value to correspond to *p *< .01 for the TD group. Had we set the |*t*| threshold of the ADHD group to correspond to the same statistical criterion, *p *< .01 (|*t*| > 2.70), the number of electrode pairs exceeding the threshold would have been influenced by the number of participants in the ADHD group as well as the strength of the effect. In this case, using the same statistical criterion where the numbers in each group differ will inflate the number of electrode pairs satisfying the statistical criteria in the larger group even if the effect size is the same or even smaller in the ADHD group. We have therefore selected a |*t*| threshold in the ADHD group that corresponds to the same effect size in the TD group. In this case, a *Z*‐score of *Z *= 0.56 for the ADHD group corresponds to a Student's *t*‐threshold |*t*| = 3.63, and with 42 participants in the ADHD group, this in turn corresponds to *p *= .0009.

A permutation test was used to determine the statistical significance of the number of comparisons where the relevant |*t*| threshold exceeded for the independent samples *t*‐test. The permutation test used was based on the approach first described by Blair and Karniski (1993) and is briefly described. For any given point in time, we initially determined the number of independent samples *t*‐tests comparing FC in the TD and ADHD groups during the CPT A‐X task that are equal to or exceed the nominated threshold |*t*| value, designated here by the symbol *N*_t0_. We then recalculated the number exceeding the |*t*| threshold after randomly allocating 42 of the 67 participants in the combined group to the ADHD group and 25 to the TD group. The number exceeding the threshold in the ith randomization is given by *N*_ti_. This process was repeated 10,000 times producing a distribution of *N*_ti_ values (*i* = 1 to 10,000) that enabled us to determine the exact probability of observing the given *N*_t0_ if the null hypothesis was correct. Where the permutation test is conducted at multiple points in time, we apply a Bonferroni correction based on the number of permutation tests conducted. A Bonferroni corrected *p*‐value based on a *p *= .05 is quoted and in the situation where a single comparison condition of *p *< .05 applies but the corrected *p* value for significance is not reached, then the comparison is deemed not significant and labeled NS.

**Figure 2:**
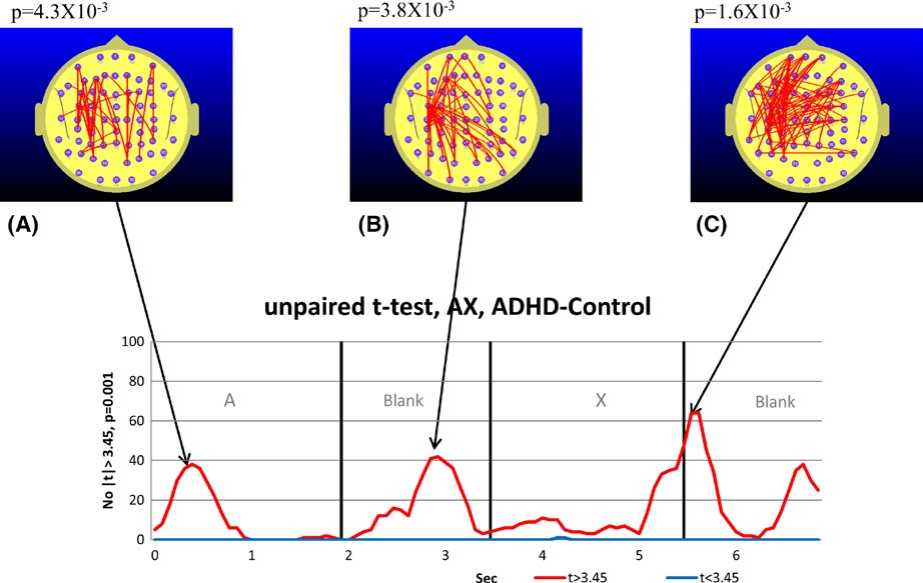
Student's *t*‐frequency graph illustrating the points in time where the number of electrode pairs where the independent samples Student's *t*‐test comparing the TD and attention‐deficit hyperactivity disorder (ADHD) group functional connectivity (FC) during the continuous performance task (CPT) A‐X task equaled or exceeded the |*t*| threshold of |*t*| = 3.45. The red trace indicates the number of electrode pairs where the Student's *t*‐test indicates that the ADHD group FC is larger than the TD group FC. The blue trace indicates the corresponding number of electrode pairs for the case where the TD group FC exceeds the ADHD group FC. A–C illustrate the FC topographic distribution and the exact *p*‐values derived from the permutation test are quoted above the maps. In this case with three permutation tests conducted, we use a Bonferroni corrected *p*‐value of 0.05/3 (*p *= .0167) to determine the relevant *p*‐value for statistical significance

**Figure 3:**
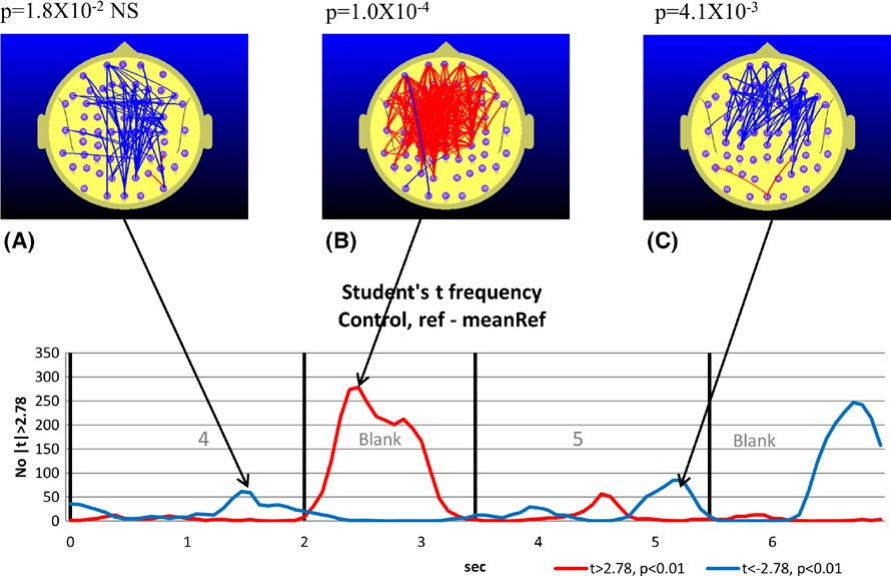
TD group. Student's *t*‐frequency graph illustrating the points in time where the number of electrode pairs where the Student's *t*‐test comparing functional connectivity (FC) during the reference task with the mean of the reference task *(meanRef*) yielded a difference significant at the *p *≤ .01 (|*t*| ≥ 2.78) level corresponding to a *Z*‐score of |*Z*| ≥ 0.56. The red trace indicates the number of electrode pairs where the Student's *t*‐test indicates that the FC during the reference task is larger than *meanRef* at the *p *≤ .01 level (*t *≥ 2.78). The blue trace indicates the corresponding number of electrode pairs where the Student's *t*‐test indicates that the FC during the reference task is smaller than meanRef at the *p *≤ .01 level (*t *≤ 2.78). A–C illustrate the distribution of electrode pairs corresponding to the points in time indicated by the arrows. The red lines indicate electrode pairs where FC during the reference task is significantly above that of *meanRef* (*p *≤ .01) and the blue lines indicate where FC during the reference task is significantly below that of *meanRef*. The lower figures in B and C illustrate the electrode pairs for the *p *≤ .01 criterion. The exact *p*‐value arising from the permutation test is indicated above figures illustrating the distribution of electrode pairs (A, B. and C). With three permutation tests conducted, we use a Bonferroni corrected *p*‐value of 0.05/3 (*p *= .0167) to determine the relevant *p*‐value for statistical significance

**Figure 4:**
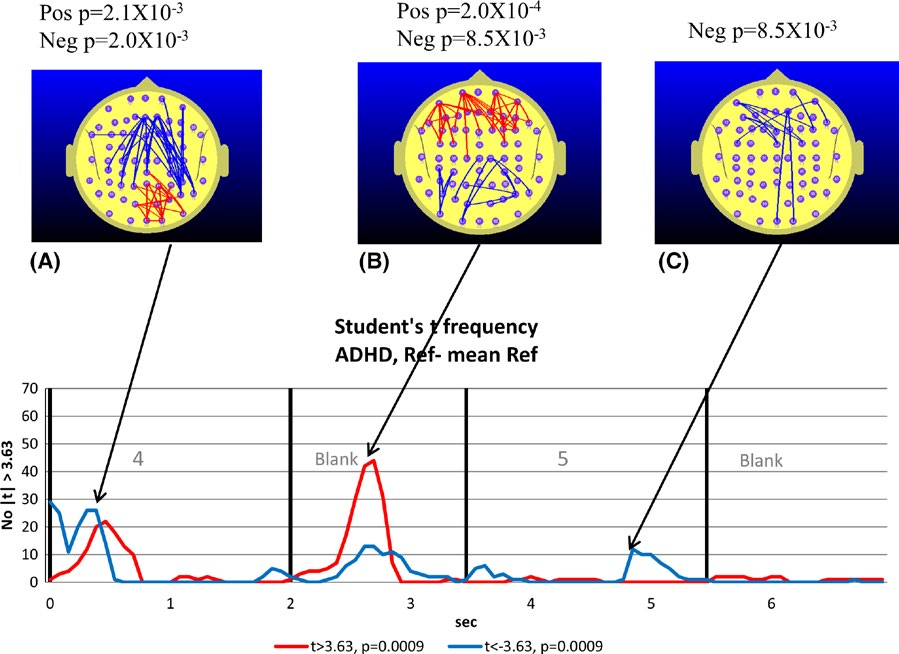
Attention‐deficit hyperactivity disorder (ADHD) group. The text describing the graph and topographic maps of functional connectivity (FC) is identical to that in Fig. 3. To control for the larger size of the ADHD group, we apply the same *Z*‐score criterion used with the TD group, i.e., |*Z*| ≥ 0.56 corresponding to *p *< .0009. With three permutation tests conducted, we use a Bonferroni corrected *p*‐value of 0.05/3 (*p *= .0167) to determine the relevant *p*‐value for statistical significance

**Figure 5:**
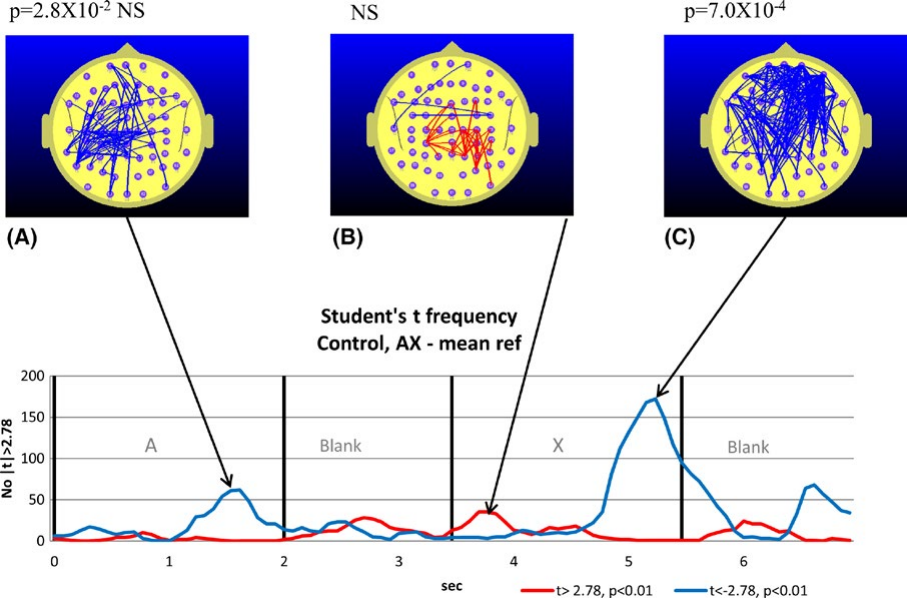
TD group. Student's *t*‐frequency graph illustrating the points in time where the number of electrode pairs where the Student's *t*‐test comparing functional connectivity (FC) during the continuous performance task (CPT) A‐X task with *meanRef* yielded a difference significant at the *p *≤ .01 (|*t*| ≥ 2.78) level. The red trace indicates the number of electrode pairs where the Student's *t*‐test indicates that the FC during the CPT A‐X task is larger than *meanRef* at the *p *≤ .01 level (*t *≥ 2.78). The blue trace indicates the corresponding number of electrode pairs where the Student's *t*‐test indicates that the FC during the CPT A‐X task is smaller than *meanRef* at the *p *≤ .01 level (*t *≤ 2.78). A–C illustrate the distribution of electrode pairs corresponding to the points in time indicated by the arrows. The red lines indicate electrode pairs where FC during the CPT A‐X is significantly above that of meanRef (*t *≥ 2.78, *p *≤ .01) corresponding to |*Z*| ≥ 0.56 and the blue lines indicate where FC during the CPT A‐X task is significantly below that of *meanRef*. The exact *p*‐value arising from the permutation test is indicated above figures illustrating the distribution of electrode pairs (A and C). With three permutation tests conducted, we use a Bonferroni corrected *p*‐value of 0.05/3 (*p *= .0167) to determine the relevant *p* value for statistical significance

For specific points in time, a permutation test was also used to determine the statistical significance of the number of comparisons where the relevant |*t*| threshold value was exceeded for the one‐sample *t*‐tests described above. This was determined separately for the positive and negative Student's *t*‐values at each of the points in time illustrated in Figs 2, 3, 4, 5 3 4 5 and was briefly described. For any given point in time, we initially determined the number of single‐valued *t*‐tests comparing FC during either the reference of CPT A‐X task with the *meanRef* that are equal to or exceed the nominated threshold |*t*| value, designated here by the symbol *N*_t0_. We then recalculated the number of comparisons exceeding the nominated threshold *N*_ti_ after swapping the *meanRef* and relevant task (reference or CPT A‐X) FC values in a random subset of the TD or ADHD group where N_ti_ is the number exceeding the threshold for the ith random subset. This process was repeated 10,000 times producing a distribution 10,000 of *N*_ti_ values (i = 1–10,000) that enabled us to determine the exact probability of observing the given *N*_t0_ in either the positive or negative direction if the null hypothesis was correct. Where the permutation test is conducted at multiple points in time, we apply a Bonferroni correction as previously described.

It should be noted that this permutation test estimation of the probability (*p*) of observing a given number of comparisons where the |*t*| threshold is exceeded takes into account the multiple comparisons made (2016) as well as the correlation between electrode pairs.

For each of the points in time where the FC distribution is illustrated, the relevant *p*‐value derived from the permutation tests is quoted.

To examine the relationship between reaction time (RT) and FC, the linear correlation between individual mean CPT A‐X RT and FC was calculated for each point in time for the ADHD and TD groups. This yielded 2016 time series illustrating the correlation between FC and RT across the whole sample. To explore temporal variation in the strength of the correlation between processing speed and FC for the TD group, we determined the number of electrode pairs where the magnitude of the correlation coefficient r exceeds 0.5, (|*r*| ≥ 0.5) a threshold value corresponding to *p *= .01 at each point in time for the TD group. The corresponding *r*‐threshold for the ADHD group is also set at |*r*| ≥ 0.5 which corresponds to *p *≤ .0007. Figures 7 and 8 comprise plots illustrating the temporal variation in the number of FC measures correlated with processing speed exceeds the *r*‐threshold are termed “correlation frequency curves”. In displaying the correlation frequency curve for both groups using the same *r*‐threshold, as opposed to matched *p*‐values, we avoid the situation where the effect strengths in the ADHD group may appear inflated because of the larger ADHD group. As with the Student's *t*‐frequency curve, a permutation test was used to determine the statistical significance of the number of correlations between RT and FC that exceeded the *r*‐threshold value of |*r*| = 0.5. We briefly describe the permutation test used to test the correlation between RT and FC. For any given point in time for the TD or ADHD group correlation frequency curve, the number FC–RT correlations equal to or exceeding the |*r*| = 0.5 threshold is determined and designated as *N*_r0_. The individual reaction times for all participants in either the TD or ADHD group are then randomized so that any given FC and RT measure are unlikely to be associated with the same individual. The number of FC–RT correlations satisfying the threshold condition is then calculated (*N*_ri_) and the process is repeated 10,000 times (*i* = 1–10,000). This enabled us to determine the probability of observing the *N*_r0_ correlations satisfying the threshold condition on the assumption that the null hypothesis applies. Where the permutation test is conducted at multiple points in time, we apply a Bonferroni correction as previously described.

## 3 Results

### 3.1 CPT‐AX behavioral data

The average RT for correct responses on the A‐X task was *M* = 495 ms, *SD* = 140 ms in the TD group and *M* = 570 ms, *SD* = 142 ms for the ADHD group. The RT for the TD group was found to be significantly faster than for the ADHD group (*F*(1, 66)  = 4.357, *p *= .041). This a common observation (Epstein et al., 2003) and will not be commented on further.

### 3.2 SSVEP‐ERPC group differences

Brain FC as reflected in the SSVEP‐ERPC was found to change according to task demands. Figure 1A illustrates the cross subject average FC for a single electrode pair FP2‐FC5 while the TD group undertook the reference task and the CPT A‐X tasks. In addition, the FC time average for the reference task (meanRef) is also represented as a heavy horizontal line. Figure 1B illustrates the equivalent electrode pair FC for the ADHD group.

While both groups exhibit temporal variations in FC that are associated with the reference and CPT A‐X tasks, the ADHD group FC appears higher than the equivalent TD group FC. However, Fig. 1 only illustrates the FC variations for one of the 2016 distinct electrode pairs. To see whether the ADHD group FC differed from the TD group FC, we used an independent samples Student's *t*‐test to examine the TD–ADHD group FC differences during the CPT A‐X task. At the threshold level selected, almost all comparisons satisfying or exceeding the threshold were associated with the ADHD group FC being larger than the TD group FC. This is illustrated in Fig. 2.

#### 3.2.1 Changes in functional connectivity during the reference task

The maps illustrated in Figs 3 and 4 indicate the location of the electrode pairs associated with each of the points in time associated with peaks in the *t‐frequency graph*. The blue lines illustrate the electrode pairs where FC during the reference task (relative to the *meanRef*) was significantly lower than *meanRef* according to a paired Student's *t*‐test. The red lines illustrate the equivalent situation where FC was significantly larger than *meanRef*.

The results of the permutation tests used to determine the statistical significance of the peak number of *t*‐tests exceeding the threshold value are listed above each map. Where the permutation test indicates the number of both positive and negative *t*‐tests are significant at the same point in time, then a separate *p*‐value is listed for the positive and negative values.

The 7.0 s interval comprising the appearance of the number “4” followed by the blank and then the target number “5” are associated with significant changes in FC for both groups. During the appearance of the “4”, we observe a nonsignificant tendency to a reduction in parieto‐frontal FC in the TD group (Fig. 3A) and a significant parieto‐frontal reduction in the ADHD group (Fig. 4A), although this occurred earlier in the ADHD group. The reduced parieto‐frontal FC in the ADHD group was also associated with an increase in occipito‐parietal FC (Fig. 4A) that was not observed in the TD group.

During the blank period between the “4” and “5”, we observe a dramatic increase in central, frontal, and prefrontal FC in the TD group (Fig. 3B). As we will be frequently referring to FC changes in the 1.5 s blank interval between the appearance of the “4” and “5”, we will refer to this interval simply as the *Ref*‐*Blank* interval. While smaller and restricted to frontal and prefrontal sites, we also observe a significant FC increase in the ADHD group during the *Ref*‐*Blank* interval (Fig. 4B). The major difference in FC topography between the groups during the *Ref*‐*Blank* period was the appearance of a reduction in FC at occipito‐parietal sites in the ADHD group Fig. 4B). Approximately 1.5 s after the appearance of the “5”, we observed a decrease in frontal and prefrontal FC in the TD group (Fig. 2D) which was also apparent in the ADHD group (Fig. 3C).

#### 3.2.2 Functional connectivity changes during A‐X Task compared to the time average of the reference task

**Figure 6:**
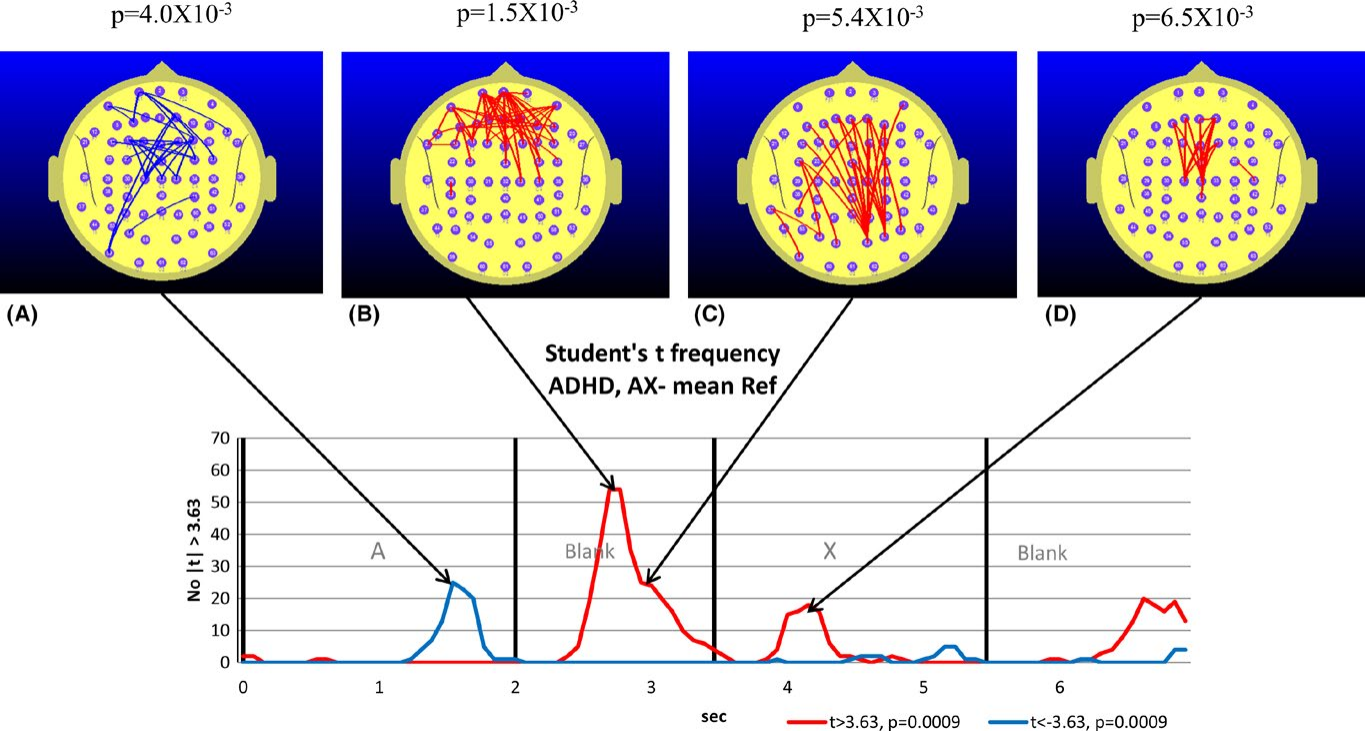
Attention deficit hyperactivity disorder (ADHD) group. The text describing the graph and topographic maps of functional connectivity (FC) is identical to that in Fig. 5. A–D illustrate the distribution of electrode pairs corresponding to the points in time indicated by the arrows. The red lines indicate electrode pairs where FC during the continuous performance task (CPT) A‐X is significantly above that of *meanRef* (*t *≥ 3.63, *p *≤ .0009) corresponding to *Z *≥ 0.56 and the blue lines indicate where FC during the CPT A‐X task is significantly below that of *meanRef* (*t *≤ −3.63, *p *≤ .0009). The exact *p*‐value arising from the permutation test based on the *p *< .0009 (*t *≥ 3.63) threshold is indicated above figures illustrating the distribution of electrode pairs (A–D). With four permutation tests conducted, we use a Bonferroni corrected *p*‐value of 0.05/4 (*p *= .0125) to determine the relevant *p*‐value for statistical significance. The major feature of note is the increased prefrontal FC during the blank between A and X changes to a parietal‐frontal component in <300 ms

The approach used to examine changes in FC during the reference task was also used to examine FC changes during the CPT A‐X task. In this case, we examined the CPT A‐X FC with respect to the time averaged reference task (*meanRef*). Figure 5 illustrates the changes in FC for the TD group while Fig. 6 illustrates the equivalent FC changes in the ADHD group.

During the appearance of the “A”, both groups exhibited reduced FC approximately 1.5 s after the appearance of the “A” although there were differences in the strength and location of the FC decreases. In the TD group, the left parieto‐frontal FC reduction approached but did not reach statistical significance (Fig. 5A). By comparison, the FC decrease in the ADHD group at this point in time was statistically stronger and located primarily at frontal and prefrontal sites (Fig. 6A).

The blank period occurring between 2.0 and 3.5 s was the time that the largest group differences in FC were observed. As we will be frequently referring to FC changes in the 1.5 s blank interval between the appearance of the “A” and “X” of the CPT A‐X task, we will refer to this interval as the *A‐X Blank* interval. While there were no differences in FC between the CPT A‐X task and meanRef that reached statistical significance in the TD group during the *A‐X Blank* period, the ADHD group exhibited a statistically robust increase in prefrontal‐frontal and parieto‐frontal FC (Fig. 6B). The FC increase seen in this interval in the ADHD group also exhibited a distinct change in topography over the period. At the 2.7 s point in time, the FC increase was predominantly apparent at prefrontal and frontal regions (Fig. 6B). Some 0.3 s later at the 3.0 s point, the ADHD group FC increase is now predominantly located at the parieto‐frontal regions (Fig. 6C).

During the appearance of the X, the TD group and ADHD group exhibited differences in FC. The TD group exhibited a small FC increase approaching significance at parietal sites approximately 250 ms after the appearance of the X (Fig. 5B) and a robust decrease in parieto‐frontal and parieto‐prefrontal FC approximately 1.8 s after the appearance of the X (Fig. 5C). By contrast, the ADHD group exhibited an increase in frontal connectivity approximately 0.7 s after the appearance of the X. This FC increase peaked shortly after the mean response time for the ADHD group. During the appearance of the X, the FC changes in the TD group and ADHD group appear to complement each other with the early FC increase being most apparent in the ADHD group (Fig. 6D) while the later FC decrease is most apparent in the TD group (5c).

#### 3.2.3 Relationship between functional connectivity and reaction time

**Figure 7:**
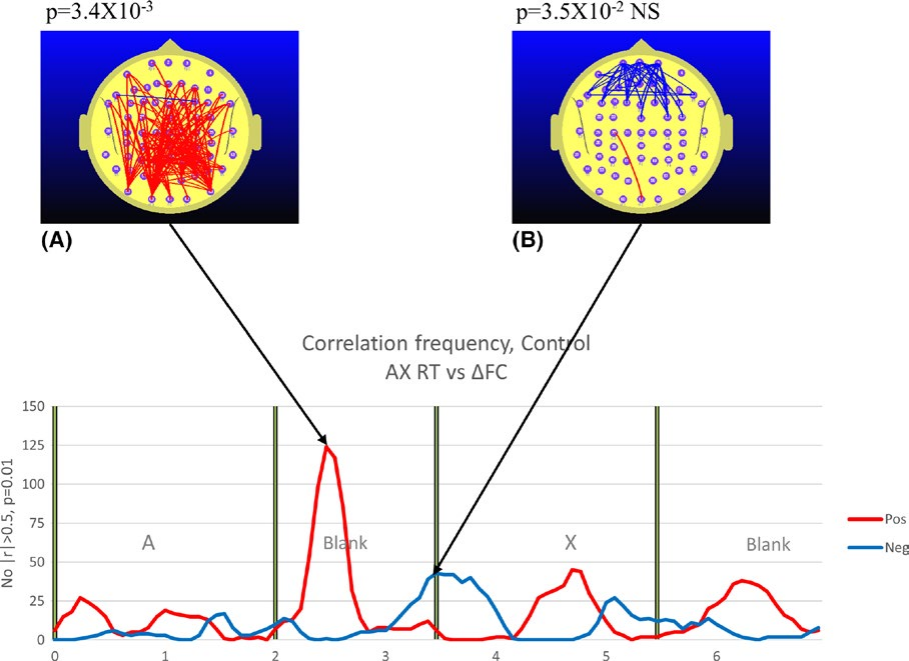
TD group. The graphs illustrate at each point in time where the number of measurements of functional connectivity (FC) during continuous performance task (CPT) A‐X minus *meanRef* FC (ΔFC) that are correlated with reaction time (RT) at the *p *< .01 (|*r*| ≥ 0.5). The red trace indicates the number of electrode pairs where ΔFC is positively correlated with RT, that is, higher ΔFC is associated with a slower individual response. The blue trace refers to the number of electrode pairs where ΔFC is negatively correlated with RT, that is, higher ΔFC is associated with a faster individual response. The most prominent feature is the robust positive correlation between parieto‐frontal ΔFC and RT. The exact *p*‐value arising from the permutation test based on the *p *< .01 (|*r*| ≥ |0.50) threshold is indicated above figures illustrating the distribution of electrode pairs (A and B). With two permutation tests conducted, we use a Bonferroni corrected *p*‐value of 0.05/2 (*p *= .025) to determine the relevant *p*‐value for statistical significance

**Figure 8:**
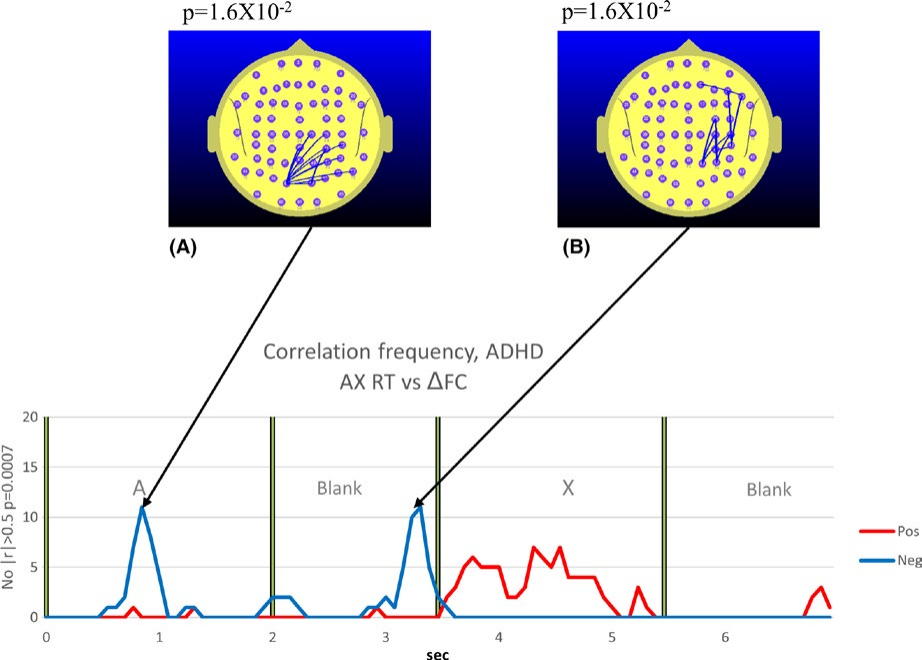
Attention‐deficit hyperactivity disorder (ADHD) group. The explanatory legend for Fig. 7 also applies to Fig. 8 for the ADHD group. The exact *p*‐value arising from the permutation test based on the *p *< .0007 (|*r*| ≥ 0.50) threshold is indicated above figures illustrating the distribution of electrode pairs (A, B)

To examine the relationship between FC and RT while performing the CPT‐AX task, we calculated the correlation coefficient between mean individual RT during successful CPT A‐X trials and the difference between CPT A‐X FC and *meanRef*. We designate the difference between CPT A‐X FC and *meanRef* by the term ΔFC. Figure 7 illustrates the number of electrode pairs where RT and ΔFC were correlated with a correlation coefficient, *r* > |0.50| corresponding to *p *< .01 for the TD group. Figure 8 illustrates the corresponding graph for the ADHD group where |*r*| ≥ 0.5 corresponding to *p *≤ .0007.

The nature of the relationship between RT and ΔFC not only varies with the group, but also with the point in time during the trial as well as the ΔFC topography. In this paper, we will focus on the RT–ΔFC relationship in both groups during the *A‐X Blank* period, the interval where we observe the most robust differences between the control and ADHD groups.

In the TD group, there are two clear and distinct patterns in the RT–ΔFC relationship. Approximately 0.5 s into the A‐X blank, there is a robust positive correlation between occipito‐frontal and parieto‐frontal ΔFC and RT (Fig. 7A). In other words, the higher this ΔFC component, the slower the mean individual response. This picture changes significantly 1.5 s into the *A‐X Blank* just as the X appears. Although only approaching significance, we see that the prefrontal ΔFC is appears to be negatively correlated with RT, that is to say, the higher the prefrontal ΔFC at the appearance of the “X”, the faster the mean individual response (Fig. 7B).

Immediately prior to the appearance of the “X” in the ADHD group, we see that the right parieto‐frontal ΔFC is negatively correlated with RT (higher right parieto‐frontal ΔFC associated with faster responses) (Fig. 8B) in the ADHD group. This correlation drops rapidly on the appearance of the “X”. So while both groups exhibited examples of higher ΔFC being correlated with faster responses immediately prior to the appearance of the “X”, the cortical regions involved differed significantly between the groups. In the TD group, it was higher prefrontal ΔFC that was correlated with faster responses, while in the ADHD group, it was the right parieto‐frontal FC that was positively correlated with faster responses.

## 4 Discussion

We found a number of significant FC differences between the TD group and ADHD group. However, as the methodology used in this study may be unfamiliar, we start this section with a restatement of some of the major findings to orient the reader before discussing the significance of the results in more detail.

- Compared to the TD group, the ADHD group exhibited elevated parieto‐frontal and temporal‐frontal FC during the CPT A‐X task that peaked during the appearance of the “A” and during the *AX Blank* period, and at the transition from the “X” to the following blank period (see Fig. 2).
- While both the TD and ADHD groups exhibited increased frontal‐prefrontal FC compared to the *meanRef* during the *Ref Blank*, the FC changes of the groups diverged during the CPT A‐X task (see Figs 3 and 4). Compared to the *meanRef*, the TD group exhibited no change in FC during the blank period between the “A” and “X” while the ADHD group exhibited significant frontal‐prefrontal and parieto‐frontal FC increases during this interval (see Figs 5 and 6).
- In the TD group, higher ΔFC in the *A‐X Blank* interval prior to the appearance of the “X” is associated with slower responses to the appearance of the target “X”.

A direct comparison of TD and ADHD group FC while performing the CPT A‐X task reveals elevated parieto‐frontal FC in the ADHD group although this effect was most apparent at certain points in time during the task. While the direct comparison between TD and ADHD groups FC during the CPT A‐X task reveals robust increases in ADHD group FC, these differences could result from different combinations of FC changes in the TD and ADHD groups. For example, the elevated ADHD group frontal FC (Fig. 2C) is most likely due to frontal‐prefrontal FC reduction in the TD group that was not apparent in the ADHD group. The subsequent analysis will thus focus on FC connectivity changes that are specific to each group before commenting further on the direct comparison.

We will discuss our FC findings in the context of possible cortical network abnormalities underlying ADHD. As such, we will draw on DMN findings as this network features prominently in recent theories of ADHD. However, it should be noted that the fMRI findings concerning the behavior of cortical networks, including the DMN in ADHD are typically described in terms of changes neural activity at various network nodes rather than changes in FC between the various network nodes (see Castellanos & Proal, 2012). By contrast, our findings refer to changes in FC rather than neural activity. Thus, while changes in FC and neural activity are distinct phenomena, we suggest they may be related in that an increase in FC between two cortical regions is generally associated with increased neural activity in these regions (see Bressler, Coppola, & Nakamura, 1993; Gregoriou, Gotts, Zhou, & Desimone, 2009; Liang, Bressler, Ding, Desimone, & Fries, 2003). Evidence for the link between increased neural activity and increased FC is most clearly observed in the behavior of cortical local field potentials recorded during a cognitive task. For example, primate studies show that increases in visual attention are associated with increased dural EEG coherence between cortical regions and these increases in coherence are associated with increased neural activity at these regions (see Bressler et al., 1993; Gregoriou et al., 2009; Liang et al., 2003). It should be stressed that we are not suggesting that our FC measure and the fMRI indication of nodal activity are the same phenomena. However, while they clearly occur on different time scales, we do suggest that they may reflect some common underlying changes in brain FC that may be apparent over a wide range of time scales.

We start with a discussion of the TD group FC findings and then use these as a stepping stone to consider the ADHD findings.

### 4.1 Typically developing group FC changes

#### 4.1.1 The parieto‐frontal FC is high during the *Ref*‐*Blank* period in the reference task and not apparent during the *A‐X Blank* period of the CPT A‐X task

One of the most striking findings of this study is the robust increase in the TD group parieto‐frontal and parieto prefrontal FC during the *Ref*‐*Blank* period. Consistent with our hypothesis is the almost complete elimination of these FC increases in the corresponding *A‐X Blank* period between the “A” serving as a cue and the subsequent “X” serving as the target for a motor response in the CPT A‐X task. We suggest that the parieto‐frontal/prefrontal FC increases during the *Ref Blank* may represent TIN and possibly DMN activity.

As this feature most prominently differentiates the TD group from the ADHD group, the discussion section will comment on this issue at greatest length. In light of the fact that we will be referring to these specific FC changes frequently in the following discussion, we will refer to the parieto‐frontal and parieto‐prefrontal FC increases occurring during the *Ref Blank* as the *Ref‐Blank FC* while the equivalent FC changes occurring in the *A‐X Blank* as the *A‐X Blank FC*. At the outset, we discount the possibility that either *Ref‐Blank FC* or the reduced *A‐X Blank FC* are due to the motor components of the response. We base this on the fact that both the reference task and the CPT A‐X task required a motor response after the blank.

The major features of the *Ref‐Blank FC* and *A‐X Blank FC* can be summarized as follows: The *Ref‐Blank FC* is prominent while the TD group performs the low‐demand reference task, and is almost completely eliminated during the equivalent epoch of the more demanding CPT‐AX task. Our data does not allow us to differentiate between two possible interpretations of these FC task‐related changes. One is that the FC changes are associated with a task‐positive TIN that may interfere with performance in the CPT A‐X task and is thus be suppressed during the CPT A‐X task. The other possibility is the FC changes are associated with task‐negative TIN such as the DMN. However, if the *Ref‐Blank FC* is a manifestation of DMN activity, one may ask why the DMN is active during the reference task as opposed to being suppressed during the reference task as well as the CPT A‐X task. One possible explanation is suggested by the “Sentinel Hypothesis”. According to the Sentinel Hypothesis, the DMN has a role in maintaining a low‐level focus of attention thereby monitoring the environment for unexpected events. Once an unexpected event is detected, higher level focused attention is brought to bear and DMN activity is suppressed. The Sentinel Hypothesis is based on the observation that in low‐demand visual vigilance tasks that require broad surveillance of the environment rather that focused attention DMN activity in increased (Gilbert, Simons, Frith, & Burgess, 2007). Mantini and Vanduffel (2013) suggest that the observation of increased DMN activity in a low‐demand visual vigilance tasks is consistent with a “Sentinel Hypothesis” for DMN (see also Buckner et al., 2008). The fact that the *Ref‐Blank FC* is prominent in the blank period immediately preceding the predictable appearance of the “5” is also consistent with the Sentinel Hypothesis in that the reference task is a low‐demand task requiring a low‐level focus of attention.

In summary, the behavior of the *Ref‐Blank FC* is consistent with what one would expect of the DMN given the Sentinel Hypothesis and/or the task preparation hypothesis.

#### 4.1.2 Reaction time in the CPT A‐X task is positively correlated with the parieto‐frontal FC during the A‐X blank period

While the virtual disappearance of the parieto‐frontal *Ref‐Blank FC* in the TD group A‐X blank period is consistent with this FC component representing a TIN, such as the DMN, the relationship between *A‐X Blank FC* and the RT during the CPT A‐X task is also consistent with this possibility. Specifically, we observed a significant positive correlation between RT during the CPT A‐X task and parieto‐frontal FC during the A‐X blank. This positive correlation between FC and RT is a feature consistent with the properties of the DMN, specifically, increased DMN activity in an attention task is associated with slower and more variable responses (Buckner et al., 2008).

This was observed in an fMRI study, Weissman, Roberts, Visscher, and Woldorf (2006) where brain activity was observed while participants performed a local/global selective attention task. They found that longer RTs were associated with reduced prestimulus activity in executive networks involving the ACC and reduced inhibition or greater activity in the DMN, including the posterior cingulate and the precuneus. These observations were confirmed in a study by Prado and Weissman (2011) who reported that a positive correlation between the PCC (a key DMN region) and the DLPFC (a key task‐positive region) was associated with a slower current response in a selective visual attention task. Consistent with the positive correlation of DMN activity and longer RTs are the observations that daydreaming and task independent thoughts are associated with greater DMN activity (Mason et al., 2007) and that response time variability is greater when the inhibitory effect of the task‐positive attentional network on the DMN is weaker (Kelly, Uddin, Biswal, Castellanos, & Milham, 2008).

While the behavior of *Ref‐Blank FC* and *A‐X Blank FC* appears consistent with that of the DMN, our data does not permit one to reliably identify this FC component as a direct manifestation of DMN activity. Without access to high‐spatial resolution brain imaging that is necessary to identify activity in specific DMN nodes, it is difficult to differentiate between a number of possible mechanisms that may account for our observations. Specifically, the *Ref‐Blank FC* and *A‐X Blank FC* may represent DMN FC changes or these may represent FC changes in cortical networks that are driven by DMN activity, or these may represent FC changes in other (non DMN) TINs that interfere with task performance and are thus suppressed during task execution. In light of this, we will refer to this parietal‐frontal FC component as *DMN‐like* FC.

### 4.2 Functional connectivity changes in the ADHD group

In comparing the FC changes in the ADHD group with those of the TD group, we will focus on the changes during the *A‐X Blank* and the *Ref‐Blank* periods. While there were a number of topographic and timing differences in FC when comparing control and ADHD groups, this discussion section will focus on two principal differences.

- The TD group exhibited a parieto‐frontal FC increase in the *Ref Blank* (Fig. 3B) that was not apparent in the *A‐X Blank* (Fig. 5). By contrast, the ADHD group exhibited the reverse. During the *Ref Blank*, only a prefrontal FC increase was observed and there was no evidence of a parieto‐frontal FC increase in the ADHD group (Fig. 4B). However, during the *A‐X Blank*, the ADHD group demonstrated a robust prefrontal and parieto‐frontal FC increase (Fig. 6B and C).
- In the ADHD group, right parieto‐temporal FC is negatively correlated with RT (Fig. 8B). This was not observed in the TD group.

It should be mentioned that these FC differences are not due to the ADHD group making more errors as we are only considering data from correct trials.

#### 4.2.1 Increased parieto‐frontal FC during *A‐X Blank* interval

While the ADHD group early FC increase in the *A‐X Blank* is restricted to the prefrontal region (Fig. 6B), the later FC increase occurring approximately 200 ms later is predominantly parieto‐frontal (Fig. 6C) and similar in topography to the parieto‐frontal FC increase that we observed in the TD group in the *Ref‐Blank* of the reference task (Fig. 3B). Furthermore, the TD group parietal‐frontal FC observed in the *A‐X Blank* is positively correlated with RT (Fig. 7A). This suggests that the later parietal‐frontal FC increase seen in the ADHD group (Fig. 6B) may represent a process similar to that observed in the TD group while they performed the reference task. Specifically, the parieto‐frontal FC increase is a manifestation of DMN‐like activity.

If this interpretation is correct, one is then left with the following question. What is the significance of the ADHD group failing to exhibit the DMN‐like transient FC increase during the reference task and then exhibiting this FC transient increase during the more demanding CPT A‐X task when it is supressed in the TD group? One interpretation of our ADHD findings is that the increased DMN‐like activity in the *A‐X Blank* interval may be playing a similar role to the increased DMN‐like activity in the *Ref‐Blank* interval in the TD group. In the TD group, we argued that the DMN‐like activity in the *Ref‐Blank* interval may have had a functional role in surveying the environment, in keeping with the Sentinel Hypothesis. It may be that the ADHD group requires a higher level of attentional demand or arousal to engage the DMN in its Sentinel role function. In other words, the TD group is able to engage brain networks appropriate to the task (DMN in low‐demand and task‐positive networks in the CPT A‐X task) while the ADHD group inappropriately engages the DMN in the more demanding CPT A‐X task. This suggestion is consistent with a large body of evidence pointing to reduced suppression of DMN activity in attentional tasks in ADHD and a positive correlation between reduced DMN suppression and distractibility in ADHD (see Castellanos & Proal, 2012).

While fMRI‐based studies of FC in ADHD have not reported task‐related increases in parieto‐frontal FC in ADHD, EEG studies have done so. Murias, Swanson, and Srinivasan (2007), reported elevated parieto‐frontal EEG coherence in the lower and upper EEG alpha band in ADHD compared to controls when participants performed a word generation task. In addition, several resting‐state fMRI FC studies of ADHD have reported reduced negative correlation between the task positive and the DMN. In an adult ADHD study, Castellanos et al. (2008) found reduced negative correlation between the ACC, a node of the executive task network and the precuneus and PCC, both nodes of the DMN. This reduced negative correlation between the DMN and the ACC and the PCC was also seen in a young cohort of 23 boys diagnosed with ADHD (Sun et al., 2012). Interestingly, the same authors also reported that the TD group exhibited increased anticorrelation between the ACC and the PCC as they aged from 11 to 15 years. By contrast, the ADHD group showed an insignificant trend to increase anticorrelation and overall exhibited a tendency to a positive correlation between the ACC and the PCC. Sonuga‐Barke and Castellanos (2007) go so far as to suggest that ADHD could be considered a “default network disorder”. Specifically, it is argued that the reduced capacity to inhibit DMN activity would be associated with increased intrusions into ongoing cognitive activity thereby compromising the ability to maintain attention to the task in hand.

If our findings offer a direct or indirect indication of DMN activity, then our data suggest that “failure to inhibit the DMN” hypothesis to account for the attention deficits in ADHD may be an oversimplification. Specifically, we see evidence of the DMN‐like parieto‐frontal activity in the low‐demand reference task in the TD group which is suppressed in the more demanding CPT A‐X task. While this feature is consistent with the fMRI studies of the behavior of the DMN, the behavior of the ADHD group shows quite a different pattern. The prominent DMN‐like parieto‐frontal FC increase seen in the *Ref‐Blank* interval in the TD group is absent in the ADHD group while it becomes very prominent in the more demanding *A‐X Blank* interval in this group. Where the “failure to inhibit the DMN” hypothesis to apply in our study, we would expect the DMN‐like parieto‐frontal FC component to be largest in the low‐demand reference task and to be reduced in the more demanding CPT AX task.

#### 4.2.2 Different FC correlates of reaction time

Our observation of a negative correlation between the right parieto‐frontal FC and RT peaking immediately prior to the appearance of the “X” (Fig. 8B) is consistent with the possibility that this right parieto‐frontal FC that is correlated with faster response times may represent what Corbetta and Shulman (2002) refers to as the “right ventral frontoparietal network”. The right ventral frontoparietal network is, as the name suggests, strongly lateralized to the right hemisphere and includes the temporal parietal junction as well as the ventrofrontal cortex. Corbetta and Shulman suggest that this network plays a critical role in directing attention to “*behaviorally relevant stimuli outside the focus of processing*.” In particular, this network is considered to be strongly stimulus driven or “bottom up” and is not engaged by any cues that carry advance information about forthcoming events *(ibid*). This is consistent with the observations of van Leeuwen et al. (1998) and McLoughlin et al. (2010) who reported abnormal preparatory states in children diagnosed with ADHD performed cued vigilance tasks such as the continuous performance task. Such preparatory state deficits would be consistent with the well‐recognized deficits in executive function associated with reduced activity at the DLPFC and dACC observed in ADHD and would also be consistent with those diagnosed with ADHD placing greater reliance on bottom‐up visual strategies in compensation for the prefrontal deficits (Bush et al., 2005; Castellanos, Sonuga‐Barke, Milham, & Tannock, 2006; Fassbender & Schweitzer, 2006).

#### 4.2.3 Network dynamics

We conclude this discussion section with some comments on the temporal dynamics of the FC patterns we have observed. If our suggestion that the parieto‐frontal FC increase seen in the *Ref‐Blank* period for the TD group and the *A‐X Blank* period for the ADHD group reflects DMN‐like activity, then it would indicate that the DMN can exhibit relatively rapid changes over the time course of <1 s. This may seem at odds with the various fMRI resting‐state findings. However, it should be appreciated that fMRI suffers significant limitations in terms of temporal resolution and the capacity to observe rapid changes in FC (Nunez & Silberstein, 2000). The fact that rapid changes in FC may be “invisible” to fMRI does not mean that these rapid changes do not occur. While fMRI does not possess the temporal resolution that would be required to observe these rapid changes (see Nunez & Silberstein, 2000) a number of studies have utilized simultaneous fMRI and EEG to examine the EEG correlates of DMN activity. One approach mentioned earlier, and reported by Helps et al. (2009, 2010) involves examining very low‐frequency EEG components, typically 0.06–0.2 Hz. The amplitude of this EEG component tends to be larger in the resting state and reduces in an attentional task. Furthermore, the attention task‐related attenuation of this component is reduced in participants exhibiting higher levels of inattention during the task, features consistent with the DMN. However, while this EEG component may reflect DMN activity as detected with fMRI, the low frequency at which the observations are made makes it impossible to examine any rapid changes in DMN activity.`

Mantini, Perrucci, Del Gratta, Romani, and Corbetta (2007) examined the relationship between fMRI measures of DMN activity and EEG over a wider frequency range of 1–80 Hz. They reported that DMN activity was correlated with EEG power variations in all of the EEG power bands (delta, theta, alpha, beta, and gamma) and that DMN activity was not exclusively reflected in any one of the EEG bands. Neuner et al. (2014) reported similar findings in a simultaneous fMRI and EEG study that used inverse EEG imaging to yield higher spatial resolution. While yielding important information on the EEG correlates of DMN activity, these types of studies were not able to shed light on the dynamics of DMN activity as the fMRI index of DMN activity was used to identify the EEG components.

In an alternative approach reported by Britz, Van De Ville, and Michel (2010), the correlation between the rapidly fluctuating EEG‐defined microstates and the fMRI‐defined resting‐state FC was examined. This yielded a significant correlation between the rapidly fluctuating EEG microstates with four of the fMRI‐defined resting‐state networks. This was in turn presented as evidence that the resting‐state FC networks are also capable of rapid change. Hayden, Smith, and Platt (2009) examined single unit and local field potentials (LFP) in the PCC, a part of the DMN while macaque monkeys performed an attention task. While they found the expected suppression of single unit activity and gamma‐frequency LFP activity during the active tasks, there was a transient increase in PCC single unit and gamma LFP at the onset of the task. Furthermore, this increased activity extended briefly into the active task. Thus, while the sustained PCC activity was consistent with the fMRI BOLD signals recorded from the DMN, the low‐temporal resolution of the BOLD signal obscured the transient increase in DMN activity at the task onset. If these primate findings also applied to humans, then the increase in DMN‐like activity can be seen in controls during the reference task, and in the ADHD group, during the control and CPT AX task can represent the equivalent transient increase in DMN activity.

Our methodology may influence the findings in two important ways. Firstly, the higher temporal resolution (Silberstein, 1995) possessed by SST will reveal rapid changes in FC not apparent with brain imaging methodologies possessing lower temporal resolution, such as fMRI and PET. However, another important factor is the 13 Hz frequency of the visual stimulus used to elicit the SSVEP. It is clear that FC is a function of frequency with high frequencies, such as the EEG gamma frequency‐mediating short‐range FC changes, while lower frequencies, such as delta‐, theta‐, alpha‐, and beta‐mediating long‐range FC changes (Bressler, 1995; Bressler & Richter, 2015; Bressler & Tognoli, 2006; Silberstein, 2006; Von Stein & Sarnthein, 2000). Thus, the frequency we have selected to examine FC will influence the FC components observed and we have to be cautious in expecting the 13 Hz FC components to precisely match the very low‐frequency FC observed with fMRI. The use of the 13 Hz SSVEP in this study has another important implication. This arises from the recent appreciation that sensory‐driven or *bottom‐up* cortical information flows are mediated by synchronous oscillations in the gamma EEG frequency range (30–90 Hz) while the internally generated cortical information flows or *top‐down* processes are mediated by synchronous oscillations in the 10–20 Hz range (Fries, 2015). Our selection of 13 Hz means that our findings are most likely preferentially sensitive to *top‐down* processes as opposed to fMRI findings that reflect very low‐frequency processes.

#### 4.2.4 Concluding comments

We have observed differences in brain FC between the control and ADHD groups. Our findings are consistent with the notion of ADHD being associated with TIN abnormalities. Specifically, our findings are consistent with the possibility of TINs in general or the DMN in particular being abnormally activated in the presence visual attention levels that were found to suppress such networks in the TD group. To the extent that our observations reflect DMN activity, then our findings call into question the simple “failure to inhibit the default mode network” hypothesis of ADHD. This is suggested in light of the fact that the TD group exhibited high levels of default mode network activity during the low‐demand reference task while the ADHD group exhibited low levels of default mode network activity during this task.

## Conflicts of Interest

The authors declare that they have no conflict of interest.
